# Mechanosensitive Piezo channels in mineralized tissues: emerging roles in osteodental adaptation and disease

**DOI:** 10.3389/fcell.2025.1607337

**Published:** 2025-07-10

**Authors:** Junchi Dong, Ran Li, Yuhuang Chen, Guixin Zhu, Xing Liang

**Affiliations:** The State Key Laboratory of Oral Diseases & National Clinical Research Center for Oral Diseases, Department of Prosthodontics, West China Hospital of Stomatology, Sichuan University, Chengdu, Sichuan, China

**Keywords:** Piezo protein, cellular mechanotransduction, bone, tooth, ion channels

## Abstract

Bone and dental tissues are highly mineralized and mechanically sensitive hard tissues. They detect and respond to mechanical forces via mechanosensitive Piezo channels, modulating physiological and pathological processes. While Piezo mechanobiology has been explored, systematic comparison of their roles across bone and dental tissues, particularly their potential crosstalk in adaptation and disease, remains underexamined in existing reviews. This review consolidates recent advances in Piezo channel biology, clarifying their structural properties, tissue-specific distribution, and functional roles in mineralized tissues. Emerging evidence highlights Piezo channels as key mechanotransducers ubiquitously expressed in skeletal and dental cellular populations. By mediating distinct mechanotransduction pathways, Piezo1 and Piezo2 modulate diverse processes, including bone remodeling, osteoblast-osteoclast communication, dental stem cell differentiation, dental hard tissue mineralization, and orthodontic tooth movement. Furthermore, their dysregulation is implicated in pathologies such as osteoporosis, pulpitis, and dentin hypersensitivity. The elucidated mechanisms establish a theoretical framework for Piezo-mediated mechanotransduction in cellular adaptation and disease progression. By integrating molecular mechanisms with regenerative applications across both osseous and dental contexts, this review advances understanding of shared mechanobiological principles in mineralized tissues and highlights translational relevance for skeletal and dental therapies. These insights align with mechanobiology and tissue engineering research, supporting future development of mechanosensitive interventions.

## 1 Introduction

Mammalian physiological hard tissues, comprising bone and dental tissues, are highly mineralized tissues with sophisticated structures that render them extremely sensitive to mechanical stimuli (Haelterman and Lim, 2019; Pei et al., 2021). This mechanosensitivity underpins a range of clinical interventions (Li et al., 2021; Shah et al., 2021)—such as distraction osteogenesis and orthodontic tooth movement—as well as pathological conditions, including osteoarthritis and traumatic periodontitis (Herrera et al., 2014; Glyn-Jones et al., 2015). Mechanotransduction denotes the cellular mechanism transducing biomechanical stimuli into intracellular biochemical signaling events (Jin et al., 2020; Jiang et al., 2021b), typically mediated through membrane depolarization or the influx of cations via mechanosensitive channels (Douguet and Honoré, 2019; Jiang et al., 2021b). These channels, which are expressed in mechanosensory organs, directly mediate cellular responses to mechanical stimuli through alterations in their physical properties (Jin et al., 2020). Existing defined mechanosensitive channels comprise the Piezo family, epithelial sodium channel/degenerin (ENaC/DEG)-superfamily proteins, TWIK-Related K^+^ Channel (TREK) subfamily proteins, transient receptor potential (TRP) polymodal receptors, transmembrane protein (TMEM) 16 superfamily, and reduced hyperosmolality-induced [Ca^2+^]_i_ increase (OSCA)/TMEM63 (Jin et al., 2020).

Notably, Piezo channels were initially characterized as non-selective cation mechanosensitive ion channels (Murthy et al., 2017), and their remarkable sensitivity is attributable to their elaborate structure. Functioning as mechanosensory receptors, Piezo channels play key roles in proprioception (Woo et al., 2015), touch (Ranade et al., 2014; Earley et al., 2022), and mechanical pain (Szczot et al., 2018; Zhang et al., 2019; Della Pietra et al., 2020), as well as in a variety of pathophysiological processes, such as inflammatory response (Solis et al., 2019), tumorigenesis and cancer progression (Jiang et al., 2022), musculoskeletal development, cardiovascular hemodynamics, renal filtration, and pulmonary homeostasis (Douguet et al., 2019; Qin et al., 2021; Xiong et al., 2022). Piezo1 and Piezo2 are the exclusive paralogs found within the mammalian Piezo channel family (Coste et al., 2010; Coste et al., 2012; Zhao et al., 2016; Murthy et al., 2017). They display no detectable sequence homology to other ion channel classes (Xiao, 2024). Predominantly localized in non-excitable cells, Piezo1 mediates intracellular Ca^2+^ signaling and activates subordinate downstream cascades to regulate diverse physiological processes (Jiang et al., 2021b). In contrast, Piezo2 is mainly distributed in excitable cells, including Merkel cells, Schwann cells, and sensory neurons (Acheta et al., 2022; Feng et al., 2022; Han et al., 2022; Sonkodi, 2022), and is indispensable for itch and pain-sensing (Feng et al., 2022), myelin formation (Acheta et al., 2022), and proprioception (Assaraf et al., 2020).

Recent studies have established Piezo channels as bona fide mechanotransducers, with growing evidence highlighting their crucial role in the mechanosensing of hard tissues. Specifically, Piezo-mediated mechanotransduction is critical for bone development and repair, stress-induced bone remodeling, toothache occurrence, and orthodontic tooth movement (OTM) (Lee et al., 2019; Jiang et al., 2021a; Qin et al., 2021; Xu et al., 2022). This review systematically introduces how Piezo1 and Piezo2 channels sense mechanical forces in bone and dental tissues, detailing their structure, activation mechanisms, distribution, and functions, thereby providing a reference framework for further investigation.

## 2 Properties of the Piezo family

### 2.1 Structural conformation

**FIGURE 1:**
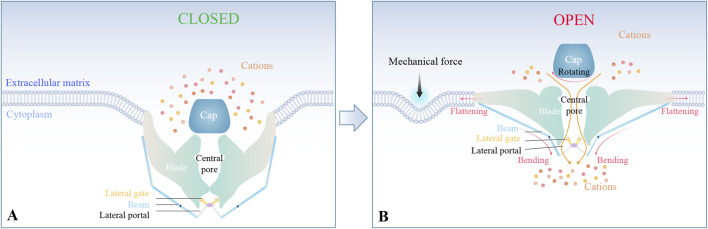
Structural basis of Piezo-mediated mechanotransduction. **(A)** Piezo1/2 adopt a propeller-shaped structure, including a central pore, an apical extracellular domain, and tripartite curved blade subunits connected to helical beams gating three lateral portals. **(B)** Under mechanical stimulation, the nanobowl-like conformation undergoes flattening, thereby mediating cation influx through the central pore and lateral portals.

Piezo1 and Piezo2 exhibit a high degree of structural homology (Jiang et al., 2021b). Both channels adopt a distinctive triskelion-like architecture consisting of a central ion-conducting pore domain, an apical extracellular dome, and tripartite curved blade subunits connected to helical beams gating three lateral portals (Ge et al., 2015; Wang et al., 2019; Yang et al., 2022) (Figure 1A).

### 2.2 Activation and inhibition mechanisms

Mechanical stimulation triggers conformational changes in Piezo channels. Specifically, the triskelion configuration distorts the bound lipid membranes into a nanobowl-like structure complex. This nanobowl-like complex flattens upon mechanical stimulation (Ge et al., 2015; Wang et al., 2019; Yang et al., 2022). The resulting flattening of the blades, coupled with the rotation of the cap and bending of the beams, facilitates cation signal transduction through the central pore and lateral portals (Ge et al., 2015; Wang et al., 2019; Yang et al., 2022). This finely tuned conformation underlies the high sensitivity of Piezo channels to mechanical forces, enabling them to modulate pathophysiological processes via cation-influx-triggered molecular signaling cascades (Figure 1B).

Piezo1 channels are gated by diverse mechanical perturbations such as poking, mechanical stretch, fluid shear stress (FSS), and hydrostatic pressure (HP), whereas Piezo2 is primarily responsive to poking and exhibits insensitivity to stretching (Sugimoto et al., 2017; Jiang et al., 2021b). Two primary paradigms govern the mechanoactivation of ion channels: the “force-from-lipids” model and the “force-from-filaments” model (Murthy et al., 2017). The former hypothesis emphasizes the mechanical energy transfer through lipid bilayer deformation under membrane interfacial tension (Arnadóttir and Chalfie, 2010). The latter hypothesis attributes stimulus transduction to molecular tethers coupling to extracellular matrix (ECM) proteins or cytoskeletal elements (Arnadóttir and Chalfie, 2010). Gating of Piezo is intimately linked to the deformations of the lipid (Cox et al., 2016; Cox et al., 2017), and cytoskeletal regulation of membrane tension may further modulate channel activity (Jin et al., 2020). Notably, the presence of the ECM enhances Piezo sensitivity, while its absence renders the channels less responsive to mechanical forces (Gaub and Müller, 2017).

Several synthetic agonists selective for Piezo1 have been identified, such as Yoda1 and Jedi1/2 (Syeda et al., 2015; Lacroix et al., 2018; Wang et al., 2018; Botello-Smith et al., 2019; Xiao, 2020). Yoda1, which is hydrophobic, activates Piezo1 possibly through a “molecular wedge mechanism,” inserting between two domains of the Piezo1 blade to promote blade extension and channel opening under subthreshold stimulation (Botello-Smith et al., 2019). However, mutations in the Piezo1 beam that abrogate Yoda1 activation suggest the involvement of additional, yet unidentified, mechanotransduction pathways (Botello-Smith et al., 2019). Moreover, Dooku1, a derivative of Yoda1, competitively blocks Yoda1-activated Piezo1-dependent Ca^2+^ signaling (Evans et al., 2018). In contrast, Piezo1 activation can be elicited by Jedi1/2, which are hydrophilic, through binding to the extracellular region of its blade structure and utilizing key mechanotransduction points in the beam (Wang et al., 2018; Xiao, 2020). Interestingly, Yoda1 and Jedi1/2 exert minimal effects on Piezo2. Nonspecific inhibitors, including GsMTx-4, FM1-43, polycationic ruthenium red (RR), streptomycin, and gadolinium, can block Piezo1/2-mediated ion signaling (Coste et al., 2010; Bae et al., 2011; Eijkelkamp et al., 2013; Xu et al., 2021). Among them, GsMTx-4 is the only known selective cation mechanical ion channel inhibitor by altering the surrounding membrane curvature (Bowman et al., 2007; Bae et al., 2011; Alcaino et al., 2017).

## 3 The position-specific function of the Piezo family in bone

**FIGURE 2:**
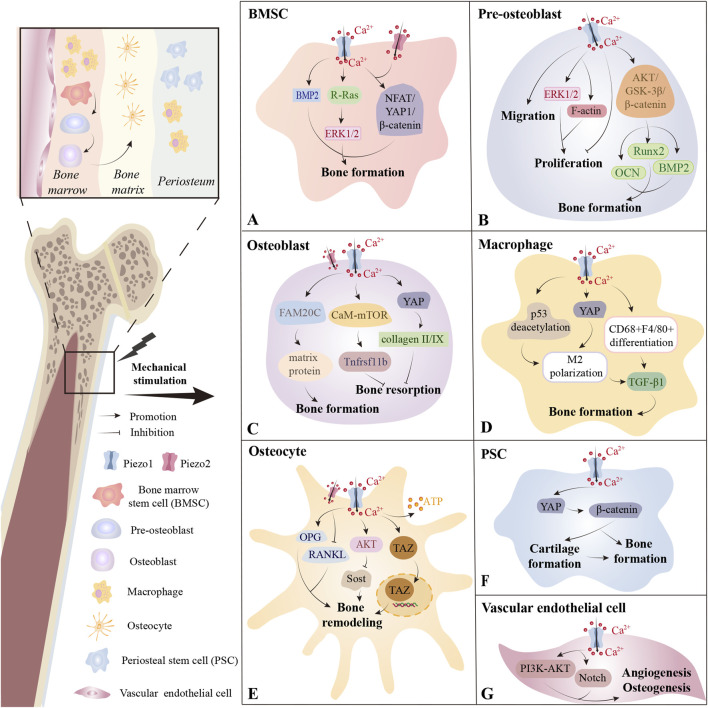
The distribution of Piezo channels across bone tissues. **(A)** In bone marrow, Piezo1 on bone marrow stem cells (BMSCs) plays a critical role in sensing mechanical stimulation and promoting bone formation through various pathways. Piezo2 may also contribute in some contexts. **(B)** Activation of the Piezo1 channel on pre-osteoblasts by LIPUS promotes cell proliferation, whereas its activation by Yoda1 inhibits proliferation. Piezo1 is also closely associated with pre-osteoblast migration and osteogenic differentiation. **(C)** In osteoblasts, Piezo1 helps maintain bone homeostasis by regulating bone formation and bone resorption. **(D)** Piezo1 on macrophages residing in both bone marrow and periosteum promotes bone formation by facilitating M2 polarization or CD68+F4/80+ differentiation and the secretion of TGF-β1. **(E)** Within the mineralized bone matrix, Piezo1 is primarily distributed on osteocytes and regulates bone remodeling. **(F)** In the periosteum, Piezo1 participates in PSC-mediated cartilage formation, bone formation, and the transition from cartilage to bone. **(G)** Piezo1 on vascular endothelial cells in bone marrow promotes angiogenesis and osteogenesis primarily through the PI3K-AKT and Notch pathways. *BMP2, Bone Morphogenetic Protein 2; ERK1/2, Extracellular Signal-regulated Kinase 1/2; NFAT, Nuclear Factor of Activated T cells; YAP, Yes-associated Protein; AKT, Protein Kinase B; GSK-3β, Glycogen Synthase Kinase 3 Beta; Runx2, Runt-related Transcription Factor 2; OCN, Osteocalcin; BMP2, Bone Morphogenetic Protein 2; FAM20C, Family with Sequence Similarity 20; Member C, CaM-mTOR; Calmodulin-Mammalian Target of Rapamycin; TGF-β1, Transforming Growth Factor Beta 1; OPG, Osteoprotegerin; RANKL, Receptor Activator of Nuclear Factor Kappa-Β Ligand; Sost, Sclerostin; TAZ, Transcriptional Coactivator with PDZ-binding Motif; PI3K, Phosphatidylinositol 3-Kinase.

Bone tissues are highly mechanosensitive and undergo adaptive remodeling in response to mechanical stimuli such as exercise and gravity (Morgan et al., 2018). Piezo1 and Piezo2 are both detected in osseous tissues, although Piezo1 exhibits a more extensive expression pattern compared to Piezo2 (Nie and Chung, 2022). Piezo1 is required to sense the biomechanical load and modulate bone formation, thereby influencing human bone mineral density (Haelterman and Lim, 2019; Bai et al., 2020). Besides, Piezo1 responds to oscillatory cortical forces by orienting and driving 3-D cell intercalations, which are important for shaping the mandibular arch in mice (Tao et al., 2019). Piezo2-mediated proprioception is essential to prevent the occurrence of skeletal deformities (Assaraf et al., 2020). Piezo channels exhibit widespread distribution across bone tissues (Figure 2).

### 3.1 In bone marrow, Piezo affects hematopoiesis and bone regeneration by mediating the mechanosensing of mesenchymal lineage cells, immune cells, and endothelial cells

Bone marrow serves as the primary site for lifelong hematopoiesis and bone regeneration, processes that are governed by interactions between bone marrow resident cells including mesenchymal lineage cells, immune cells, hematopoietic stem/progenitor cells, endothelial cells, and neuronal cells (Baccin et al., 2020). The Piezo family, particularly Piezo1, is widely distributed across mesenchymal lineage cells, immunocytes, and endothelial cells within bone marrow niches, mediating physiological functions including bone regeneration and hematopoiesis.

#### 3.1.1 Piezo family in mesenchymal lineage cells affects bone regeneration

In neonatal mice, specific deletion of Piezo1 in osteoblastic mesenchymal progenitor cells causes impaired osteoblast function and increased bone resorption, resulting in multiple spontaneous fractures (Zhou et al., 2020). Piezo1-mediated mechanotransduction is vital for the anti-aging maintenance of peri-arteriolar osteogenic progenitors and the bone morphogenetic protein 2 (BMP2) upregulation-related osteoblastic differentiation of bone marrow mesenchymal stem cells (BMSCs) (Sugimoto et al., 2017; Shen et al., 2021) (Figure 2A). In BMSCs, Piezo1/2 mediate mechanotransduction by synergistically activating nuclear factor of activated T cells/Yes-associated protein/beta catenin (NFAT/YAP1/β-catenin) (Zhou et al., 2020), and the mechanosensing function of Piezo1 has even been exploited in wearable pulsed triboelectric nanogenerator designed to facilitate bone repair (Wang et al., 2022). Recent evidence also implicates Piezo1 in activating the extracellular signal-regulated kinase 1/2 (ERK1/2) signaling cascade within BMSCs, with Piezo1’s C-terminal R-Ras binding domain critically regulating osteoblastic differentiation (Sugimoto et al., 2023).

Piezo1 deficiency in pre-osteoblasts and osteoblasts also causes reduced bone mass and spontaneous fractures (Zhou et al., 2020; Hendrickx et al., 2021). Mechanical loading parameters critically influence Piezo1-dependent responses: exposure to low-intensity pulsed ultrasound (LIPUS) enhances MC3T3-E1 pre-osteoblast proliferation via Piezo1 activation, which promotes phosphorylation of ERK1/2 and polymerization of F-actin around the nucleus—effects reversed by genetic silencing of Piezo1 (Zhang et al., 2021) (Figure 2B). In contrast, Yoda1-induced Piezo1 activation inhibits the proliferative capability of MC3T3-E1 (Yoneda et al., 2019), indicating that different modes of Piezo1 activation can produce divergent cellular outcomes. Besides, Piezo1 silencing independently inhibited the migratory capacity of MC3T3-E1 (Yan et al., 2019). Furthermore, Piezo1 is closely linked to osteogenic differentiation and matrix protein secretion (Song et al., 2020; Dzamukova et al., 2022; Kong et al., 2022), as is demonstrated by its role in nanotube-stimulated osteogenesis in MC3T3-E1 cells (Kong et al., 2022) and in upregulating osteogenic genes (e.g., runt-related transcription factor 2 (Runx2) (Song et al., 2020), BMP2, and osteocalcin (OCN) (Kang et al., 2024)) via pathways such as protein kinase B/glycogen synthase kinase 3 beta/β-catenin (AKT/GSK-3β/β-catenin) (Song et al., 2020). Besides, centrifugation force upregulates family with sequence similarity 20, member C (FAM20C) production in osteoblasts via Piezo1, leading to matrix protein secretion that modulates vascular conversion and enhances bone mineralization (Dzamukova et al., 2022) (Figure 2C).

Analogous to its role in osteogenesis, Piezo1 also critically regulates bone resorption dynamics. Piezo1-deficient mice also exhibit elevated bone resorption and resistance to unloading-induced resorption, a phenomenon that may stem from Piezo1-mediated regulation of osteoclastic differentiation through YAP-driven secretion of collagen II/IX from osteoblasts (Wang et al., 2020). However, selective deletion of Piezo1 in osteoclasts does not impact murine skeletal mass, implying that Piezo1-mediated regulation of bone homeostasis is likely independent of osteoclasts (Wang et al., 2020). Moreover, Piezo1-mediated calcium ion/calmodulin/mammalian target of rapamycin (Ca^2+^/CaM/mTOR) signaling in osteoblasts and osteocytes suppresses osteoclast formation via regulation of Tnfrsf11b expression, underscoring its protective role against age-related bone loss (Li et al., 2023). Besides, the increase of Piezo1 levels in hematopoietic progenitor cells is irrelevant to the property of applied wall shear stresses (osteoprotective or osteodestructive) (Bratengeier et al., 2020). The above studies suggest that Piezo1 affects osteoclastic activity primarily by osteoblast-osteoclast crosstalk rather than direct actions on osteoclasts and hematopoietic progenitor cells.

Osteoporosis, clinically defined as reduced bone mineral density, microstructural degradation, and elevated fracture susceptibility (Rachner et al., 2011), is alleviated by weight-bearing exercise linked to bone mechanosensitivity (Pagnotti et al., 2019). Osteoporotic patients exhibit markedly decreased Piezo1 protein levels, which correlate positively with key osteogenic differentiation biomarkers (alkaline phosphatase (ALP), OCN, and collagen type I alpha 1 (COL1A)) (Sun et al., 2019). Conditional knockout of Piezo1 within osteochondral lineages further demonstrates that loss of Piezo1 results in impaired skeletal microarchitecture, diminished mechanical integrity, and increased susceptibility to spontaneous fractures, highlighting its importance in trabecular bone formation (Sun et al., 2019). Besides, piezoelectric micro-vibration mitigates osteoporosis induced by estrogen loss via Piezo1 promotion in osteoblasts (Wu et al., 2021).

#### 3.1.2 Piezo1 in immune cells and endothelial cells affects hematopoiesis and bone regeneration

Macrophage Piezo1-YAP signaling axis activation promotes angiogenesis and osteogenesis by inducing M2 polarization (Tang et al., 2021) (Figure 2D). Changes in the physical microenvironment are caused by irradiation exerting mechanical stretch stimulation on residual bone marrow macrophages (BM-Mφs), thereby upregulating Piezo1 and activating the calcineurin/NFAT/hypoxia-inducible factor-1 alpha (HIF-1α) pathway (Zhang et al., 2022). This cascade enhances the expression of Vascular growth factor A (VEGF-A), which is a key factor for hematopoiesis (Zhang et al., 2022). Piezo1 also influences the function and glucose metabolism of bone-marrow-derived dendritic cells under tension (Chakraborty et al., 2021). Deletion of Piezo1 in bone vasculature impairs angiogenesis and osteogenesis via phosphatidylinositol 3-kinase (PI3K)/AKT and Notch signaling pathways (Chen et al., 2021) (Figure 2G).

### 3.2 In mineralized bone matrix, Piezo1 is involved in osteocyte-mediated bone remodeling

Osteocytes encased in mineralized matrix serve as primary mechanosensory cells in bone tissues (Haelterman and Lim, 2019). Piezo1 vitally functions in osteocyte sensation of FSS, supported by *in vivo* evidence showing that modulation of Piezo1 alters the load-dependent bone formation (Li et al., 2019; Sun et al., 2019) (Figure 2E). Under mechanical loading, osteocytes balance bone remodeling by adjusting the receptor activator of nuclear factor kappa-Β ligand (RANKL)/osteoprotegerin (OPG) ratio and sclerostin/dickkopf-related protein 1 (Sost/Dkk1)-mediated Wnt pathway (Nakashima et al., 2011; Robling and Bonewald, 2020; Karthik and Guntur, 2021). *In vitro*, MLO-Y4 osteocytes sense FSS through Piezo1, accompanied by upregulation of the bone formation factor OPG and downregulation of the bone resorption factor RANKL (Liu et al., 2022b). The mechanically induced Sost expression suppression in osteocytic cell line IDG-SW3 is abrogated by Piezo1 deficiency or inhibition and AKT inhibitors, suggesting that the Piezo1/AKT pathway may mediate this regulatory process (Sasaki et al., 2020). Collectively, Piezo1 regulates the transcription of critical osteogenic and osteoclastic markers of osteocytes and is fundamental to mechanosensitive osteocyte-mediated bone remodeling. Additionally, MLO-Y4 cells can detect stretching forces via Piezo1, which triggers calcium influx, transcriptional coactivator with PDZ-binding motif (TAZ) nuclear translocation, and ATP production—events that amplify BMSCs’ osteogenic capacity and may offer novel intervention strategies for mechanical bone remodeling (Ru et al., 2024).

### 3.3 In periosteum, Piezo1 coordinates bone formation through stem cell migration/differentiation and macrophage-mediated osteoprogenitor recruitment

High levels of Piezo1 expression are detected in periosteal stem cells (PSCs) as well as macrophages in the periosteum (Deng et al., 2022; Liu et al., 2022a) (Figure 2F). Studies have consistently demonstrated that Piezo1 is indispensable for PSC-mediated chondrogenesis, bone formation, and cartilaginous bone transformation during fracture repair (Liu et al., 2022a). This role may be attributed to Piezo1’s ability to enhance the migratory, osteogenic, and pro-angiogenic capacities of PSCs, primarily via the YAP/β-catenin pathway activation (Liu et al., 2022a). During the meniscal regeneration process, biomechanical stimulation triggers Piezo1-mediated Ca^2+^ influx, subsequently activating calcium/calmodulin-dependent protein kinase (CaMK) and nuclear factor of activated T-cells, cytoplasmic 1 (NFATc1), thus promoting the YAP/phosphorylated Smad2/3 (pSmad2/3)/SRY-related HMG-box 9 (SOX9) pathway (Yan et al., 2023). However, the mechanisms by which Piezo1 modulates cell migration and pro-angiogenic secretion of VEGF-A remain to be fully elucidated.

CD68^+^ macrophages are the most mechanosensitive type of macrophage in the periosteum (Deng et al., 2022) (Figure 2D). Mechanical loading activates Piezo1 in CD68+F4/80- macrophage subsets, driving their differentiation into the CD68+F4/80+ phenotypes (Deng et al., 2022). These differentiated macrophages secrete transforming growth factor beta 1 (TGF-β1) and thrombospondin-1 (Thbs1, a cytokine activating TGF-β1 by phosphorylating Smad2/3) to recruit osteoprogenitor cells (Deng et al., 2022). Additionally, mechanical strain-induced Ca^2+^ influx triggers p53 post-translational modification through coordinated acetylation/deacetylation dynamics (Cai et al., 2023). These epigenetic reprogrammings drive macrophage polarization toward an M2 reparative phenotype, enabling TGF-β1 secretion that stimulates BMSCs recruitment, clonal expansion, and osteogenic differentiation (Cai et al., 2023).

### 3.4 In periosteal nerve, Piezo2 mediates proprioception to maintain the normal development of bone tissue

In mammals, Piezo2 serves as the primary mechanotransducer for proprioception (Woo et al., 2015). Loss of Piezo2 function in humans leads to prenatal proprioceptive impairment, triggering abnormalities in joint positioning and ultimately leading to bone disorders like hip dysplasia, scoliosis, and distal arthrogryposis (Coste et al., 2013; McMillin et al., 2014; Chesler et al., 2016; Delle Vedove et al., 2016; Haliloglu et al., 2017; Mahmud et al., 2017; Uehara et al., 2020). A study in mice further confirmed that selective Piezo2 deficiency in proprioceptive neurons—but not in chondro-osteoprogenitor lineages—resulted in skeletal malformations such as aberrant hip and spinal structure (Assaraf et al., 2020).

## 4 Piezo family in tooth tissue

**FIGURE 3:**
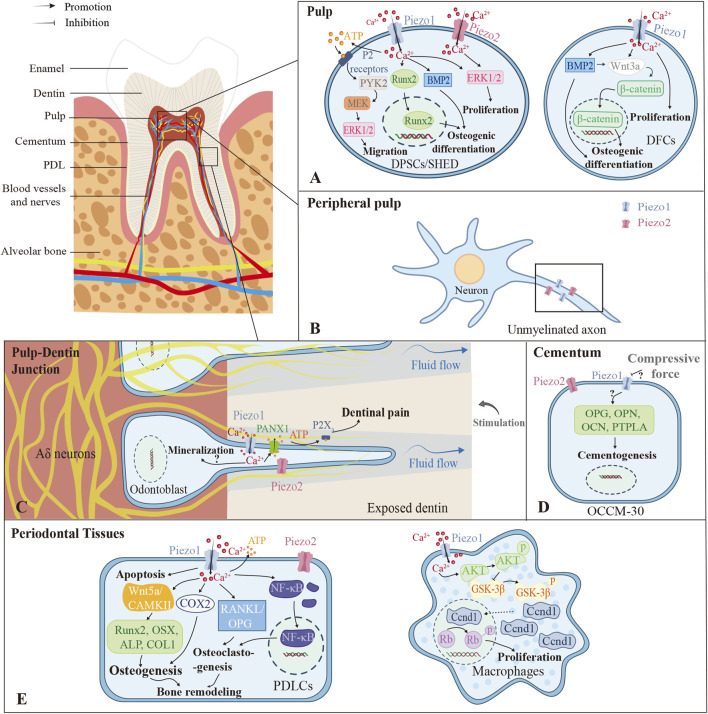
Different roles the Piezo channels play in different parts of the tooth. **(A)** The pulp contains stem cells regularly. Piezo channels in DPSCs and SHED can promote migration, osteogenic differentiation, and proliferation through various pathways with mechanical stimulation. DFC-expressed Piezo1 promotes cell proliferation and osteogenic differentiation capacity primarily through β-catenin-mediated Wnt3a signaling. **(B)** In peripheral pulp, Piezo1/2 are mainly localized in unmyelinated axons. **(C)** The Piezos are closely related to dentin sensitivity, mainly via the Piezo1-PANX1-P2X3 axis. But whether Piezo1/2 promote dentin mineralization still remains controversial. **(D)** In cementum, compressive force downregulates Piezo1 expression and cementogenic markers in OCCM-30, while some studies show opposite results. **(E)** In periodontal tissues, Piezo1/2 in PDLCs affect cell apoptosis, osteogenesis, osteoclastogenesis, and bone remodeling. Piezo1 also exerts positive effects on the proliferation of macrophages through the Piezo1-AKT-Ccnd1 pathway. *PDL, Periodontal Ligament; DPSCs, Dental Pulp Stem Cells; SHED, Human Exfoliated Deciduous Teeth; DFCs, Dental Follicle Cells; PTPLA, Protein Tyrosine Phosphatase-like Protein-A; PDLCs, Periodontal Ligament Cells; PYK2, Protein Tyrosine Kinase 2; Runx2, Runt-related Transcription Factor 2; MEK, Mitogen-activated Protein Kinase; ERK1/2, Extracellular Signal-regulated Kinase 1/2; BMP2, Bone Morphogenetic Protein 2; PANX1, Pannexin-1; OPG, Osteoprotegerin; OPN, Osteopontin; OCN, Osteocalcin; CAMKII, Calcium/Calmodulin-dependent Protein Kinase II; COX2, Cyclooxygenase-2; OSX, Osterix; ALP, Alkaline Phosphatase; COLI, Collagen Type I; RANKL, Receptor Activator of Nuclear Factor Kappa-Β Ligand; NF-κB, Nuclear Factor Kappa-Β; AKT, Protein Kinase B; GSK-3β, Glycogen Synthase Kinase 3 Beta; Ccnd, Cyclin D.

Similar to bone tissue, Piezo channels in dental tissue regulate cell differentiation and pathological processes by sensing mechanical forces, but their distribution and function are tissue-specific. Tooth tissues consist of the inner pulp and the outer hard tissues including dentin, enamel, and cementum. The cementum is connected to the periodontal tissues to support the tooth. While prior research has delineated Piezo channel localization within the pulp, dentin, and periodontal tissues, their distribution in other dental compartments and involvement in various dental-related pathophysiological activities remain to be further investigated. Figure 3 shows the distribution of Piezo in dental tissues and the related downstream pathways (Figure 3).

### 4.1 In dental pulp, Piezo affects the proliferative activity and differentiation capacity of pulp-derived stem cells and is involved in pulpal pain perception

Dental tissues harbor various stem cell populations, exemplified by dental pulp stem cells (DPSCs) and stem cells from human exfoliated deciduous teeth (SHED), both demonstrating self-renewal and multipotency—attributes that make them promising for tissue engineering (Zhai et al., 2019) (Figure 3A). DPSCs are sensitive to mechanical stimulations, which promote their proliferation while reducing the viability and adhesive properties (Gaite et al., 2024). Yoda1-induced activation of Piezo1 enhances migration of human DPSCs (hDPSCs) via an ATP-dependent protein tyrosine kinase 2 (PYK2)/mitogen-activated protein kinase (MEK)/ERK signaling cascade (Mousawi et al., 2020), while mechanical or chemical activation of Piezo1 promotes osteogenic differentiation by modulating BMP2 expression (Sugimoto et al., 2017). Static pressure can increase the expression of Piezo2 in hDPSCs as well (Kang et al., 2024). In SHED, Piezo1 regulates the nuclear translocation of Runx2, which is essential for osteoblast and odontoblast differentiation (Miyazaki et al., 2019). Additionally, in dental follicle cells (DFCs), which are contributors to cementogenesis, periodontal ligament formation, and alveolar bone development (Zhou et al., 2019), Piezo1 activation via Wnt3a/β-catenin signaling promotes proliferation and osteoblastic lineage commitment (Xing et al., 2022) (Figure 3A).

Pulp pain is mediated by slow-conducted unmyelinated C-fibers and fast-conducted myelinated A-fibers (Bender, 2000). In pulpitis, inflammatory mediators lower the nociceptor thresholds, activating pain-associated ion channels (Yang et al., 2024). Piezo channels may contribute to the initial hyperemic response, potentially via Piezo2-mediated vascular mechanotransduction, given its detection in the blood vessel walls of human dental pulp (Gaite et al., 2024).

Piezo1 predominantly drives inflammatory progression, localizing to small myelinated Aδ fibers (60.2%), large myelinated Aβ fibers (24.3%), and unmyelinated C fibers (15.5%) (Bryniarska-Kubiak et al., 2024). Its expression increases progressively during irreversible pulpitis and correlates significantly with pro-inflammatory cytokines (interleukin-1beta (IL-1β), IL-6, tumor necrosis factor-alpha (TNF-α)) (Yang et al., 2024).

Piezo2 serves as the primary mechanonociception transducer, functioning as a low-threshold mechano-detector (Wan et al., 2024) enriched in Merkel cells and myelinated afferents (Ohyama et al., 2022). Critically, in peripheral pulp, both channels localize to unmyelinated axons ascending toward dentin, indicating their roles in mediating acute mechanical pain (Figure 3B) (Cho et al., 2022; Han et al., 2022). Piezo2 specifically facilitates glutamate release via vesicular transporters (Cho et al., 2022; Han et al., 2022). During irreversible pulpitis, Piezo2 downregulation occurs despite its strong association with pain mediators (neuropeptide Y (NPY), substance P, Tachykinin 1 (TAC1)) and overall pain intensity (Yang et al., 2024). Mechanistically, the cAMP signaling pathway potentiates Piezo2 mechanosensitivity in inflammation (Yang et al., 2024), consistent with its role in inflammatory mechanical hyperalgesia (Wan et al., 2024).

Collectively, although both Piezo1/2 are expressed on pulp nerve fibers and function as mechanosensitive channels directly involved in mediating pulp pain, they exhibit functional divergence: Piezo1 amplifies inflammatory responses while Piezo2 directly mediates nociception and may participate in vascular hyperemic responses. Their inverse expression dynamics highlight distinct pathophysiological roles.

### 4.2 In dentin, Piezo mediates the perception of pain and is associated with dentin formation

In rodents, Piezo2 is predominantly expressed in mature odontoblasts (Khatibi Shahidi et al., 2015), while Piezo1 is primarily localized to the cell membrane and cytoplasm of human and murine odontoblasts (Huang et al., 2024). However, Gaite et al. (2024) proposed an alternative expression pattern in human teeth, demonstrating that Piezo1/2 are mainly present in pre-odontoblasts rather than mature odontoblasts and are absent in dentinal tubules. In contrast, murine odontoblasts exhibit widespread Piezo1/2 immunoreactivity, particularly at the basal pole (Gaite et al., 2024). These discrepancies are likely attributed to technical variations, highlighting the need for further investigation into Piezo channel localization in human odontoblasts.

Dentin sensitivity (DS) refers to pain arising from exposed dentin, not attributable to other dental diseases, and is best explained by hydrodynamic theory (Mantzourani and Sharma, 2013). According to this theoretical framework, external stimuli increase dentinal tubular fluid efflux, generating hydrodynamic shear stress on mechanosensory nerves in the tubules and then activating the Aδ nerve at the pulp-dentin junction, eventually leading to pain (Mantzourani and Sharma, 2013). Researchers demonstrate that Piezo1 and TRPV1/2/3/4 channels function as mechanosensors in this process, facilitating pannexin-1 (PANX1)-dependent ATP secretion, thereby establishing a communication pathway between odontoblasts and sensory neurons (Ohyama et al., 2022) (Figure 3C). Dental pain is triggered by P2X_3_ receptor activation due to extracellular ATP release (Sato et al., 2018). Pharmacological blockade of the Piezo1/TRPA1-PANX1-P2X_3_ axis in odontoblasts significantly reduces cold-induced pain responses in exposed dentin (Ohyama et al., 2022).

Additionally, Huang et al. (2024) revealed that Piezo1 promotes odontoblast mineralization *in vitro* via Ca^2+^/PI3K-AKT/semaphorin 3A (SEMA3A) by way of inducing hDPSCs to differentiate into odontoblasts, and they further confirmed its involvement in reactive dentin formation *in vivo*. Conversely, Matsunaga et al. (2021) identified that chemically activated Piezo1 inhibits the mineralization of odontoblasts, whereas knockdown of Piezo1 promotes the mineralization, and that Piezo1 is also vital for the suppression of dentinogenesis after cellular deformation within dentin tubules. These findings suggest that Piezo1 may exert context-dependent effects on odontoblast mineralization, warranting further investigation.

### 4.3 In cementum, Piezo1 affects the cementogenic activity of cementoblasts

The cementum, which covers the root dentin and provides the anchor for the periodontal ligament (PDL) (Foster, 2017), is primarily composed of a mineralized matrix secreted by cementoblasts and collagen fibers derived from the PDL (Nuñez et al., 2019). The ability of cementoblasts to secrete mineralized matrix makes them pivotal in the formation of restorative cementum and the reconstruction of periodontal function (Nuñez et al., 2019). An *in vitro* study using the murine cementoblast model OCCM-30 confirmed the Piezo1 expression and found that the knockdown of Piezo1 exacerbated the decrease in the expression of cementogenic activity markers caused by static mechanical force (Zhang et al., 2017) (Figure 3D). Additionally, micro-CT imaging of Piezo1-knockout mice revealed marked reductions in cellular cementum, alveolar bone volume, and cementum ECM mass (Zhang et al., 2023), but this may result from diminished periodontal ligament stem cells (PDLSCs) differentiation into cementoblasts. In contrast, HP has been shown to increase Piezo1 expression in cementoblasts while suppressing cell migration and OPG expression, indicating that compressive forces impair cementoblast function by enhancing Piezo1 activity (Wang et al., 2023). Therefore, the precise role of Piezo1 in human cementogenesis and the underlying mechanisms involved remain to be further investigated.

### 4.4 In periodontal tissues, Piezo mediates periodontal tissue remodeling due to orthodontic tooth movement

OTM denotes the therapeutic application of controlled biomechanical forces to reposition misaligned dental units through coordinated periodontal remodeling (Jiang et al., 2021a). This biological process specifically involves structural adaptation of the periodontium components: alveolar bone, periodontal ligament (PDL), and gingiva (Jiang et al., 2021a). During OTM, the remodeling of tissues is triggered by the mechanical signal transduction of periodontal ligament cells (PDLCs) and osteocytes (Li et al., 2021). PDLCs exhibit dual expression of Piezo1 and Piezo2, with Piezo1 being more abundant (Horie et al., 2023). However, RR-mediated Piezo suppression has no effect on the proliferation of PDLCs (Gao et al., 2017). Activation of Piezo1 by Yoda1 promotes periodontal tissue regeneration through the stimulation of Lepr^+^ periodontal ligament stem cells (PDLSCs) (Zhang et al., 2023) and converts mechanical stimuli into intracellular calcium influx that modulates downstream signaling cascades such as Notch, ERK, nuclear factor kappa-Β (NF-κB), and so on (Jiang et al., 2024).

In a rat OTM model, Piezo1 activation on the tension side boosts osteogenic markers (Runx2, osterix (OSX), ALP, and collagen type I (COL1)) and elevates osteoclastic activity, both fundamental for alveolar bone remodeling (Jiang et al., 2021a) (Figure 3E). The non-canonical Wnt/Ca^2+^ pathway could be associated with this process, as indicated by correlations between Wnt5a/CAMKII expression and bone-related molecules on the tension side (Du and Yang, 2023). An *in vitro* study also showed that Piezo1 expression was upregulated in PDLCs after stretch loading, and this upregulation was closely related to stress-stimulated transcriptional activation of cyclooxygenase-2 (COX2) coupled with modulation of RANKL/OPG signaling axis in PDLCs (Shen et al., 2020). Furthermore, Piezo1 expression can be enhanced by mechanical tensile force in human PDLSCs (hPDLSCs), thus activating the Notch1 pathway and facilitating their osteogenic differentiation capacity (Lin et al., 2020).

Recent studies manifest that Piezo1 has an impact on pressure-triggered PDLC apoptosis (Jiang et al., 2024) and inflammatory gene expression (Schröder et al., 2023), while also promoting osteoclast differentiation (Zheng et al., 2024). Under mechanical pressure, Piezo1 activation in PDLCs elevates the expression of pro-inflammatory genes (TNF, IL-6, prostaglandin-endoperoxide synthase 2 (PTGS2)) (Schröder et al., 2023), upregulates pro-apoptotic proteins (Bax and caspase-3), and inhibits anti-apoptotic proteins, thereby promoting apoptosis via the p38/ERK1/2 pathway (Shen et al., 2023). Importantly, in the compression areas of OTM models, upregulated Piezo1 and β-catenin can be detected (Jiang et al., 2024), accompanied by increased RANKL/OPG ratios (Zheng et al., 2024). Inhibition of Piezo1 reduces the distance of tooth movement (Horie et al., 2023; Jiang et al., 2024). Interestingly, Piezo1 appears to mediate osteoclastogenesis in a context-dependent manner—upregulating RANKL under pressure while downregulating OPG in the absence of mechanical strain (Schröder et al., 2023). This may involve distinct intracellular signaling pathways, calling for further exploration. Additionally, Piezo1 on the PDLC membrane facilitates extracellular ATP release under compressive force, a mechanism that is critical for both bone remodeling and pain perception during orthodontic treatment (Horie et al., 2023).

Furthermore, immune cells including macrophages are recruited and play an integral regulatory role during OTM (Li et al., 2021). Early recruitment of M1 macrophages initiates osteoclastogenesis, whereas later recruitment of M2 macrophages suppresses osteoclastic activity and promotes bone deposition (Li et al., 2021). The Piezo1/AKT/cyclin D (Ccnd1) axis is essential for the proliferation and infiltration of macrophages in periodontal tissues during OTM (Xu et al., 2022) (Figure 3E). GsMTx4 also indirectly encourages the osteolytic differentiation of RAW264.7 by affecting the NF-κB pathway in periodontal ligament cells (Jin et al., 2015). Nevertheless, it remains enigmatic whether Piezo1 mediates macrophage polarization during OTM.

## 5 Discussion

As mechanotransducers, Piezo1/2 channels convert mechanical stress into cation influx, activating downstream signaling pathways that regulate diverse pathophysiological processes. Piezo1 and Piezo2, activated by different mechanical and chemical stimuli, are widely distributed in bone and teeth tissues. The cell-type-specific expression patterns and functional roles of Piezo channels across these mineralized tissues are systematically summarized in Table 1.

**TABLE 1:** Essential functions of Piezo channels in mineralized tissues.

Tissue	Cell type	Piezo1	Piezo2	References
Bone marrow	BMSC	• Bone formation ↑ (essential role)	• Bone formation ↑ (redundant with Piezo1)	Zhou et al. (2020)
	Pre-osteoblast	• Proliferation (↑ by LIPUS, ↓ by Yoda1) • Migration ↑ • Bone formation ↑	-	Yan et al. (2019), Yoneda et al. (2019), Song et al. (2020), Zhang et al. (2021), Dzamukova et al. (2022)
	Osteoblast	• Bone formation ↑ • Bone resorption ↓	• Osteoblast differentiation ↑	Wang et al. (2020), Zhou et al. (2020), Dzamukova et al. (2022)
	Macrophage	• Angiogenesis ↑ • Osteogenesis ↑ • Hematopoiesis ↑	-	Tang et al. (2021), Zhang et al. (2022)
	Vascular endothelial cell	• Angiogenesis ↑ • Osteogenesis ↑	-	Chen et al. (2021)
Bone matrix	Osteocyte	• Bone remodeling ↑	• Bone formation ↑ (redundant with Piezo1)	Nakashima et al. (2011), Karthik and Guntur (2021), Nie and Chung (2022), Ru et al. (2024)
Periosteum	PSC	• Chondrogenesis ↑ • Bone formation ↑	-	Liu et al. (2022a)
	Macrophage	• Bone remodeling ↑	-	
	Proprioceptive neuron	-	• Proprioception	Woo et al. (2015)
Dental pulp	DPSC/SHED	• Migration ↑ • Osteogenic differentiation ↑ • Odontoblast differentiation ↑	• Proliferation ↑	Sugimoto et al. (2017), Mousawi et al. (2020), Huang et al. (2024), Kang et al. (2024)
	DFC	• Proliferation ↑ • Osteogenic differentiation ↑	-	Xing et al. (2022)
	Sensory neuron	• Inflammation ↑	• Pain transduction	Yang et al. (2024)
Dentin	Odontoblast	• Mineralization (conflicting reports: ↑/↓)	• Pain transduction	Matsunaga et al. (2021), Ohyama et al. (2022)
Cementum	Cementoblast	• Cementogenesis (conflicting reports: ↑/↓)	• Expressed (role unclear)	Nottmeier et al. (2023), Wang et al. (2023)
Periodontal tissue	PDLC	• Orthodontic remodeling ↑ • Inflammation ↑ • Apoptosis ↑	• Minor contribution	Jiang et al. (2021a), Horie et al. (2023), Schröder et al. (2023), Jiang et al. (2024)
	Macrophage	• Proliferation ↑	-	Xu et al. (2022)

“↑” indicates promotion, “↓” indicates inhibition, and “-” indicates no report.

*BMSC, Bone Marrow Stem Cell; LIPUS, Low-intensity Pulsed Ultrasound, PSC, Periosteal Stem Cell; DPSC, Dental Pulp Stem Cell; SHED, Stem Cells from Human Exfoliated Deciduous Teeth; DFC, Dental Follicle Cell; PDLC, Periodontal Ligament Cell.

Building upon this comprehensive synthesis, in bone, Piezo channels are localized to mesenchymal cells, immune cells, and osteocytes within osteo-microenvironments, mediating hematopoiesis and skeletal regeneration/remodeling. In dental tissues, Piezo channels in the pulp, dentin, cementum, and periodontal tissues influence cell differentiation, proliferation, and migration and are closely associated with pulpitis and DS-induced pain, dentin/cementum mineralization, and periodontal adaptation during OTM.

Despite the progress, several unresolved questions still persist. For example, most current studies are largely confined to cellular or animal models, leaving the precise localization of Piezo channels in human hard tissues contentious. Beyond that, conflicting conclusions exist regarding Piezo-regulated dentin/cementum formation under identical mechanical force, potentially due to experimental techniques and conditions limitations, calling for the necessity for advanced techniques and standardized experimental models to reconcile context-dependent outcomes. Also, variations in force magnitude, duration, or other subtle factors may lead to the completely opposite effect that Piezo channels have on the same objects, which needs deeper investigations.

Mechanobiological understanding of Piezo channels in osseous and dental tissues could pave the way for innovative approaches in tissue engineering and disease treatment. Future studies should be based on the existing studies to clarify Piezo channels’ precise functional roles and relative mechanisms in the physiopathological processes of bone and teeth.
